# Defining a standards framework for ophthalmology real-world data methodologies through an expert-led Delphi consensus

**DOI:** 10.1038/s41433-026-04424-1

**Published:** 2026-04-02

**Authors:** Pierre-Henry Gabrielle, Francesco Bandello, Aude Couturier, Christiana Dinah, Amanda Downey, Alessandro Invernizzi, Arshad M. Khanani, Jorge C. P. Rocha, Insaf Saffar, Javier Zarranz-Ventura, Rishi P. Singh

**Affiliations:** 1 Department of Ophthalmology, Dijon University Hospital, Dijon, France; 2 Department of Ophthalmology, Vita-Salute University, Scientific Institute San Raffaele, Milan, Italy; 3 Ophthalmology Department, Foundation Rothschild Hospital, Université Paris Cité, AP-HP, Lariboisière Hospital, 2. Rue Ambroise Paré, Paris 75010, France; 25-29. Manin, Paris, France; 4 London Northwest University Healthcare NHS Trust, London, UK; 5 F. Hoffmann-La Roche Limited, Basel, Switzerland; 6 Eye Clinic, Department of Biomedical and Clinical Science, Luigi Sacco Hospital, University of Milan, Milan, Italy; Save Sight Institute, University of Sydney, Sydney, Australia; 7 Sierra Eye Associates, Reno, Nevada, USA, University of Nevada, Reno School of Medicine, Reno, NV USA; 8 iRetina Eye Institute, Salvador, Brazil; 9 Hospital Clínic of Barcelona, University of Barcelona, Barcelona, Spain; 10 Cleveland Clinic Martin Hospitals, Cleveland Clinic Florida, Stuart, FL USA

**Keywords:** Retinal diseases, Outcomes research

## Abstract

**Background/Objectives:**

Real-world data (RWD) is becoming increasingly important in ophthalmology, offering insights into clinical outcomes, therapeutic approaches, and healthcare practices. However, methodological variability limits comparability and generalisability across RWD studies. This Delphi consensus aimed to establish expert agreement on the need for standardised methodologies in ophthalmology RWD studies, identify the key clinical and patient-reported data elements that should be collected, and explore strategies for consistent implementation.

**Methods:**

A modified Delphi methodology was followed. A steering committee (SC) of three ophthalmologists developed 38 consensus statements across five key topics. These statements were developed into an online four-point Likert scale survey and distributed to healthcare professionals experienced in managing retinal diseases via members of The Ophthalmology Network. Consensus was defined a priori as ≥75% agreement. Results were shared with the SC and key recommendations were discussed.

**Results:**

A total of 244 responses were received, predominantly from retina specialists (*n* = 232, 95%), with broad representation across six regions, the largest being Europe (*n* = 116, 48%). Consensus was achieved for all 38 statements, with 36 (95%) reaching ≥90%. These statements covered key principles, including: variability of current standards, ideal clinical standards for RWD collection, RWD analysis methodology, ideal patient-reported standards, and implementing and reporting consistent standards/frameworks. As the stopping criteria were met, no further Delphi rounds were conducted. Eight key recommendations were developed.

**Conclusions:**

The outputs from this consensus aim to guide future ophthalmology RWD studies towards improved consistency, reliability, and generalisability, ultimately strengthening the evidence base for clinical decision-making to improve patient outcomes.

## Introduction

Real-world data (RWD) plays an important role in ophthalmology, bridging the gap between randomised controlled trials (RCTs) and routine clinical practice [1, 2]. Sources of RWD include electronic health records (EHRs), registries, claims databases, and patient-reported outcome measures (PROMs) [3]. As RWD includes diverse patient populations, including those often excluded from RCTs, it helps assess the broader applicability of trial findings. When analysed, these data generate real-world evidence (RWE) which informs clinical decision-making, treatment utilisation, and healthcare policy [4–6]. RWD has been valuable in optimising treatment strategies for conditions including neovascular age-related macular degeneration (nAMD) and diabetic macular oedema (DMO), particularly in supporting the adoption of treat-and-extend regimens [7, 8]. Given the importance of RWD, appropriate capture and analysis are essential to advance how ophthalmologic conditions are managed and treated.

Several guidelines exist to standardise RWD collection and reporting. The International Consortium for Health Outcomes Measurement (ICHOM) provide guidance on the minimum set of outcome measures for macular degeneration and cataract surgery that should be collected and reported as RWD [9, 10]. These guidelines aim to encourage collaborative and consistent measurement of patient outcomes to allow comparisons between care providers and countries. While not specific to ophthalmology or RWD, the Consolidated Standards of Reporting Trials (CONSORT) checklist is widely used to improve RCT reporting and can be adapted to guide RWD methodology and reporting [11]. However, despite available guidance, several challenges must be addressed for RWD to be successful in transforming the field of ophthalmology [5, 12, 13].

A major challenge is the lack of standardised methodologies specific to RWD collection. Aggregating and comparing data from different clinics is difficult due to variability in how patient information (e.g., visual acuity (VA), intraocular pressure (IOP), and optical coherence tomography (OCT) scans) is collected, and differences in EHR systems and data formats [14–17]. Variations in inclusion/exclusion criteria for RWD studies and endpoint reporting further complicate data harmonisation [3]. A systematic review identified significant heterogeneity between RWD sources; most documented patient baseline status, clinical outcomes, and treatment responses, but few reported safety records, patient-reported burden, or economic data. Only 10 of the 64 analysed sources were considered robust enough for analysis and inclusion in RWD studies [4]. In addition, while VA and treatment complications are commonly recorded in registries, long-term disease control and anatomic outcomes are less frequently reported [9]. Overall, the lack of standardised methodologies for RWD makes it challenging to compare studies, draw reliable conclusions, and ascertain data quality, thereby limiting the generation of robust RWE.

To address these gaps, the ophthalmological community requires a minimum set of standardised methodological criteria to improve the quality and consistency of RWD studies. This study aimed to establish global expert consensus on the need for standardised methodologies for ophthalmology RWD studies, identify the key clinical and patient-reported data elements that should be collected, and explore strategies for consistent implementation. A modified Delphi method was chosen to obtain consensus from a group of experts using a formally recognised method, while mitigating the risks of group biases affecting the overall decision-making process [18].

## Methods

The project aims and scope were designed by a steering committee (SC) of three retina specialists on behalf of The Ophthalmology Network, who identified the need to standardise RWD methodologies. The Ophthalmology Network is a professional international network sponsored and facilitated by Roche, comprising members from across the global eye care community, who collaboratively develop initiatives to improve patient outcomes. Roche provides organisational support, while scientific governance and opinions are member-led and do not necessarily reflect the views of Roche [19].

**Fig. 1:**
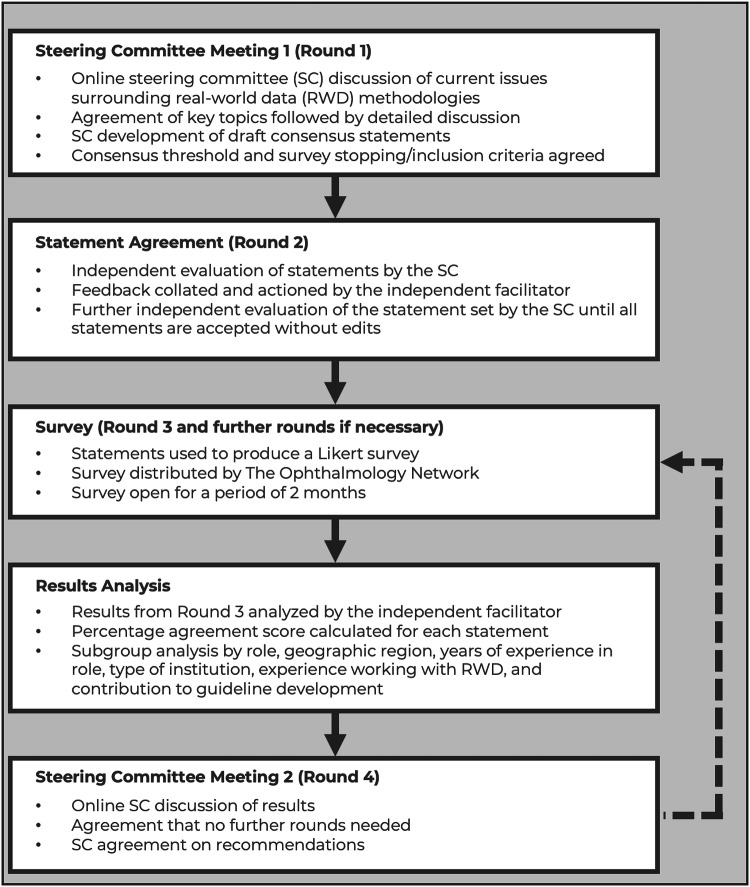
Modified Delphi study design.

A modified Delphi methodology was followed (Fig. 1), guided by an independent facilitator (Triducive Partners Limited). The study was not registered, and all reporting followed the ACCORD guidelines [20].

In March 2024, a brief literature review was conducted on existing RWD standards in ophthalmology using PubMed and a general web search. Search terms included “ophthalmology AND real-world data”. The search was not disease-specific, and search terms were deliberately broad to assess the scope of research available. The review was used to generate pre-meeting questions for the SC, focused on challenges in current RWD collection standards.

### SC meeting 1 (round 1)

Guided by the independent facilitator, the SC convened virtually in April 2024. The results of the pre-meeting survey were discussed, and based on the responses, the SC agreed upon five topics of focus:

1. Variability of current standards (awareness, burden, unmet need)
2. Ideal clinical standards for RWD collection
3. Ideal clinical standards for RWD methodology
4. Ideal patient-reported standards
5. Implementing & reporting consistent standards/frameworks

The SC collaboratively developed 41 draft consensus statements in line with each topic. Stopping criteria were established a priori as a 2-month window to collect responses, a target of 70 responses, 90% of statements passing the threshold for consensus, and a threshold for consensus set at ≥75% (a widely accepted threshold [21]). These criteria were established to gain the required number of responses while accounting for time pressures within international healthcare systems.

### Statement agreement (round 2)

The 41 initial consensus statements were collated by the facilitator and independently rated by the SC following the meeting. As a result, 7 were removed, 11 were modified, 4 were added, and 23 were agreed for inclusion without modification. Recommendations were actioned by the facilitator, and the list of statements was re-sent to the SC for a second independent review; following this, all statements were accepted, resulting in a final set of 38 statements.

### Survey (round 3)

The 38 statements were developed into a four-point Likert scale survey (‘strongly disagree’, ‘tend to disagree’, ‘tend to agree’, and ‘strongly agree’) and distributed by The Ophthalmology Network (as described above) to a wider panel of peers. Inclusion criteria were that respondents must be employed in a relevant role (retina specialist, general ophthalmologist, or ophthalmology researcher) and have experience in managing retinal diseases. The survey was distributed across Africa, Asia, Australia/Oceania, Europe, and North and South America. No translations were undertaken, and all communications were undertaken in English.

### Results analysis

Responses were captured using Microsoft Forms. A statement of consent was included at the start of the survey, and consent was implied through survey completion and submission. No incentives were offered. Anonymity was planned in the study design: no personal information or protected characteristics beyond role, geographic region, years of experience in role, type of institution, experience working with RWD, and contribution to guideline development were captured. Neither the SC nor the facilitator knew the respondent identities. As no patient-specific data were involved, ethical approval was not sought. Completed surveys were analysed by the independent facilitator using Microsoft Excel. For each statement, responses indicating agreement (“tend to agree” and “strongly agree”) were combined and expressed as a percentage of total responses to calculate overall agreement. Statements meeting the predefined consensus threshold were considered to have achieved consensus.

### SC meeting 2

In January 2025, the SC and additional Ophthalmology Network members (n = 6) convened to review the results. The combined SC agreed that while the stopping criteria had been met, the results showed a bias towards Europe. To address this, the survey was redistributed in North and South America for one month. The combined SC used the remainder of the meeting to identify the key statements for each topic and develop draft recommendations. Draft recommendations were independently reviewed and amended by the SC and ophthalmology network during the process of manuscript development, following review of the updated results.

## Results

A total of 244 responses to the survey were received and included in the final analysis. Respondents were predominantly retina specialists (*n* = 232, 95%) (Supplementary Fig. 1) and had over five years of experience in their role (*n* = 227, 93%) (Supplementary Fig. 2). Responses were received from across six regions, with the largest responder group being from Europe (*n* = 116, 48%) (Supplementary Fig. 3). Full demographic data is presented in Supplementary Figs. 1–6.

**Fig. 2:**
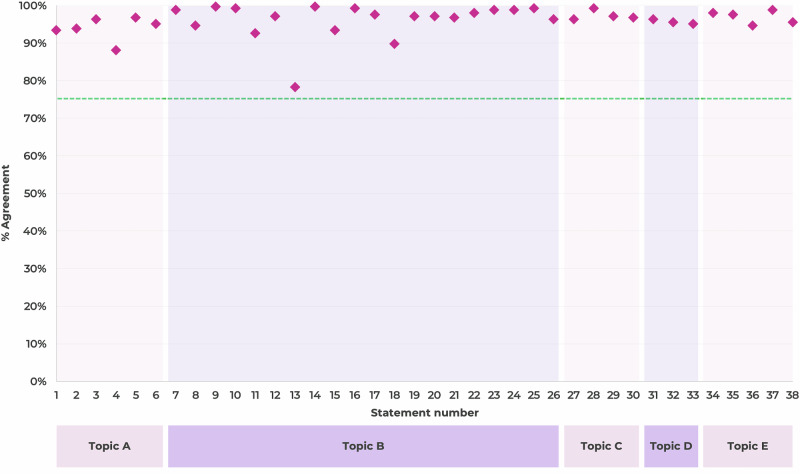
Consensus agreement levels by statement. The threshold for consensus is depicted by the green dashed line (75%). Statements are grouped by topic as follows: Topic A, variability of current standards (awareness, burden, unmet need); Topic B, ideal clinical standards for real-world data (RWD) collection; Topic C, ideal clinical standards for RWD methodology; Topic D, ideal patient-reported outcome standards; and Topic E, implementation and reporting of consistent standards and frameworks.

The consensus threshold of ≥75% agreement was achieved for all 38 statements, with 36 (95%) reaching ≥90% agreement. The statements and corresponding agreement levels are presented in Table 1 and illustrated in Fig. 2. As the stopping criteria were satisfied, no additional testing rounds were conducted. The SC discussed the results and agreed on a set of eight recommendations to help guide future ophthalmology RWD studies towards improved consistency, reliability, and generalisability.

**Table 1:** Defined consensus statements and corresponding levels of agreement.

No:	Statement	Agreement Score
**Topic A. Variability of current standards (awareness, burden, unmet need)**		
1.	The variability in standardised data collection and methodology for IVT therapies in neovascular age-related macular degeneration (nAMD) represents a gap	**93%**
2.	The variability in standardised data collection and methodology for IVT therapies in diabetic macular oedema (DMO) represents a gap	**94%**
3.	An unmet need exists regarding the standardisation/utilisation of baseline data collection in nAMD and DMO	**96%**
4.	An unmet need exists regarding the standardisation of assessments (e.g. OCT) to measure treatment outcomes across nAMD and DMO	**88%**
5.	The absence of standardised protocols in RWD collection and methodologies underscores the need for standardised data collection and methods to improve reliable and generalizable research findings	**97%**
6.	The lack of interoperability between electronic medical records is a systematic barrier limiting the standardisation of RWD collection	**95%**
**Topic B. Ideal clinical standards for RWD collection**		
7.	Data collection methodologies should be standardised across different healthcare settings, regions, and platforms to enable seamless integration and comparison of data from diverse sources	**99%**
8.	Implementing a single point of entry for RWD collection in nAMD and DMO is crucial for streamlining data acquisition, enhancing data quality, and promoting interoperability	**95%**
9.	There should be ongoing evaluation and optimisation of RWD standards to incorporate advancements in technology, research methodologies, and healthcare practices, ensuring that RWD remains relevant and impactful in informing clinical decision-making and healthcare policy	**100%**
10.	There should be a minimum set of standards for recording patient baseline characteristics for real-world evidence studies	**99%**
11.	Inclusion and exclusion criteria for real-world evidence studies should be standardised to improve reliable and generalizable research findings	**93%**
12.	For diabetic patients, HbA1c measurements and current medications should be collected as part of RWD collection standards in DMO	**97%**
13.	Intraocular pressure (IOP) represents an important clinical standard for RWD collection in nAMD and DMO	**78%**
14.	There should be an aligned approach on recording treatment outcomes in RWE studies, including a combination of anatomical and functional parameters	**100%**
15.	Visual acuity is a primary clinical outcome for RWD in nAMD and DMO, providing a reliable and universally recognised measure of visual function	**93%**
16.	Anatomical parameters serve as an important clinical standard for RWD collection in nAMD and DMO	**99%**
17.	Retinal fluid measurements, such as IRF and SRF, are important core data variables for RWD collection in nAMD and DMO	**98%**
18.	Central subfield thickness (CST) is an important endpoint for RWD collection in DMO	**90%**
19.	Comorbidities such as previous cataract surgery, previous vitrectomy, and fellow eye disease or status should be collected as part of RWD collection standards in nAMD	**97%**
20.	Comorbidities such as hypertension, hypercholesterolemia, kidney disease, myocardial infarction, stroke, and peripheral vascular disease should be collected as part of RWD collection standards in DMO	**97%**
21.	RWD core variables should include demographic data, e.g., age, gender, ethnicity, socioeconomic status	**97%**
22.	RWD collection standards should include the intended type of dosing and the intervals between treatments and why this duration was chosen	**98%**
23.	To account for real-world variability in patient scheduling, methodological standardisation should also include a framework on how to measure treatment intervals and treatment delays / non-adherence / non-persistence	**99%**
24.	RWD collection standards should include the number of treatments provided	**99%**
25.	RWD collection standards should include ocular adverse events during the treatment period (whether at administration or afterwards)	**99%**
26.	RWD core variables for DMO should include information about diabetes history, such as time since diabetes diagnosis, type of diabetes, mean HbA1c levels, trends in fasting glucose levels, and systemic therapies (e.g. insulin therapy and aGPL1)	**96%**
**Topic C. Ideal clinical standards for RWD methodology**		
27.	Fostering collaboration among stakeholders, including clinicians, researchers, patients, and data administrators, is essential to develop consensus-driven standards for RWD studies that reflect the diverse needs and perspectives of the healthcare community	**96%**
28.	Transparency regarding the methodology and criteria used to define study cohorts is essential for ensuring the reproducibility, reliability, and credibility of cohort studies in nAMD and DMO	**99%**
29.	Including all inclusion and exclusion criteria for patients included in RWD studies is essential for ensuring transparency, reproducibility, and reliability in nAMD and DMO research	**97%**
30.	RWD studies should represent patients in an unbiased way and not exclude patients based solely on baseline vision	**97%**
**Topic D. Ideal patient-reported standards**		
31.	Patient-Reported Outcome Measures (PROMs) complement clinical outcomes and provide information on how the disease and treatment burden can impact patients’ lives, guiding more patient-centred care	**96%**
32.	Including PROMs at baseline and then every 12 months in nAMD and DMO provides valuable insights into patients’ symptoms, treatment satisfaction, and overall experiences with care	**95%**
33.	Collecting PROMs at baseline and then every 12 months throughout the course of a study helps identify areas for improvement in eye care, assess intervention effectiveness, and tailor treatment to meet patients’ needs, enhancing overall healthcare outcomes and experiences	**95%**
**Topic E. Implementing & reporting consistent standards/frameworks**		
34.	Providing education, training, and support to researchers, clinicians, and other stakeholders on best practices in RWD collection and methodology is essential for ensuring consistency, adherence to standards, and quality in RWD research and analysis	**98%**
35.	Transparent reporting of RWD methods, including detailed descriptions of study design, data sources, patient selection criteria, outcome measures, and data analysis techniques, is essential for facilitating peer review, validation, and replication of study findings	**98%**
36.	To ensure transparent reporting of RWD collection methods, journals should require the submission of standardised checklists (such as those provided by ICHOM and CONSORT)	**95%**
37.	Regular evaluation and refinement of RWD collection and methodology standards, based on emerging evidence and best practice, is essential to adapt to evolving healthcare landscapes and optimise the utility of RWD in informing clinical practice and improving patient outcomes	**99%**
38.	Open access to the full anonymised dataset in RWD is critical for fostering transparency, promoting collaboration, and advancing knowledge in nAMD and DMO	**95%**

## Discussion

The results from this consensus confirm that the ophthalmological clinical community agrees there is a need to standardise RWD methodologies. The near-unanimous agreement across all statements highlights that establishing a standard framework for ophthalmology RWD methodologies is an important step toward improving the quality of research, supporting clinical decision-making, and enabling more reliable assessment of treatment outcomes.

### Topic A: variability of current standards (awareness, burden, unmet need)

#### Recommendation 1

*Standardised protocols for data collection in ophthalmology need to be developed to help improve the reliability and generalisability of RWD*

The consensus achieved on the statements in Topic A highlights the unmet need for standardised data collection and methods to improve the reliability and generalisation of RWD in ophthalmology. The high agreement on Statements (henceforth ‘S’) 1–4 (88–96%) emphasised substantial variability in the way data is collected and the methodologies followed regarding IVT therapies for nAMD and DMO, but also in baseline data collection and treatment outcome assessment measures. This aligns with a study of 64 RWD sources from 16 countries in retinal disease that found substantial variation in data collection, including recording baseline characteristics, treatment regimens, and outcome measures[4]. This reinforces the view that more standardised and structured methodologies for RWD are needed. Moreover, the lack of interoperability between electronic medical records (S6; 95%) was recognised as a systemic barrier to consistent data capture, which is a widely acknowledged challenge [22]. Overall, the consensus achieved for Topic A highlights the need for a standardised framework to guide consistent and clinically meaningful collection and reporting of RWD in ophthalmology.

### Topic B: ideal clinical standards for RWD collection

#### Recommendation 2

*A collective effort is needed to develop single-point data entry healthcare systems to help facilitate integration and comparison of data*

#### Recommendation 3

*A minimum set of standards for recording patient characteristics at baseline and follow-up assessments is needed. These should include:*

- A mix of anatomical and functional parameters
- Demographic data, e.g., age, gender, ethnicity, socioeconomic status
- Ocular adverse events during the treatment period (whether at administration or afterwards)
- Treatment intervals (explicitly capturing the number of injections and the number of patient visits)

The results from Topic B provide a strong foundation for defining the ideal clinical standards for RWD collection in ophthalmology. These recommendations align with and extend the existing ICHOM minimum outcome set for macular degeneration, which similarly emphasises the importance of capturing functional parameters and demographic data [9]. This consensus builds on these foundations by incorporating treatment-related variables (e.g., intervals, dosing rationale, adverse events) and extending the scope to other disease contexts beyond macular degeneration.

The consensus achieved for S8 (95%) highlights the importance of implementing a single-point data entry system to streamline RWD acquisition, enhance data quality, and promote interoperability. This is particularly relevant given that current EHR systems are often perceived as complex and time-intensive; physician documentation time was shown to increase from 16 to 28% following EHR implementation[23]. Initiatives such as the Fight Retinal Blindness! (FRB) registry capture and analyse RWD on treatment outcomes and safety for retinal diseases. This registry is structured and quick to complete; however, it is a web-based platform that is not embedded within EHR systems or routine clinical records, leading to additional data entry burden[24]. Therefore, there is a need for a single-point entry system that can be integrated into routine practice to minimise duplication of effort. However, implementing such systems globally faces significant barriers, including incompatible EHR platforms, high costs, and limited technical infrastructure. In this context, pilot implementations within individual centres or health systems may reduce duplicate data entry and demonstrate practical feasibility, thereby informing and encouraging broader adoption.

Findings from this topic also identified that core demographic data, including age, gender, ethnicity, and socioeconomic status (S21; 97%), should be collected to support equity-focused analyses and subgroup comparisons. Treatment-related information should also be consistently recorded, including the intended dosing regimen, treatment intervals, the rationale for interval selection (S22; 98%), the total number of treatments provided (S24; 99%), and any ocular adverse events observed during or following treatment (S25; 99%). Emphasis should be placed on recording ocular adverse events, as this is critical to ensure real-world safety data is captured and to identify complications not always seen in clinical trials.

To aid in the recording of RWD, methodological standardisation should include a framework for measuring treatment intervals, delays, non-adherence, and non-persistence, accounting for real-world variability in clinical practice (S23; 99%). However, there is often a difference between planned treatment schedules and actual treatment delivery in clinical practice. A retrospective chart review study analysed 4,857 anti-VEGF injections across 518 eyes and revealed that 21.1% of anti-VEGF injections were administered more than one week later than intended, and at a patient level, nearly half (49.4%) experienced treatment delays exceeding seven days; longer delays also trended with worse VA outcomes[25]. This demonstrates that real-world practice often diverges from planned schedules, highlighting the clinical importance of capturing treatment adherence patterns. However, requiring clinicians to document all of these aforementioned parameters may be overly burdensome. A more pragmatic approach could be to record two dates: the planned treatment date and the actual treatment date. Researchers can then calculate treatment delays, adherence rates, and persistence metrics retrospectively from these dates, without adding complexity to clinical documentation. This simplified data capture maintains analytical value while remaining feasible in busy clinical practices.

This study also defined the disease-specific clinical parameters that should be collected for RWD, pertaining to nAMD and DMO. A combination of anatomical and functional parameters should be collected; VA should be collected as the primary functional outcome, along with IOP, and retinal fluid measures such as intraretinal fluid (IRF) and subretinal fluid (SRF) (S13-17; 78–100%). S13 (78%) on measuring IOP achieved the lowest agreement amongst all consensus statements. A plausible explanation is that, while IOP is an important parameter for overall ocular health, it is not as directly related to disease activity in nAMD or DMO in the same way that IRF or SRF are [26].

Studies that include both anatomical and functional parameters provide a more comprehensive view of patient outcomes and treatment effectiveness [27]. However, guidance on RWD collection must be practical and applicable across different healthcare settings. For example, while retinal fluid measures are valuable, they require specialised software, potentially limiting the number of centres that can participate in a study [28–30]. Moreover, the same parameter may be measured using different methods or devices. Therefore, advanced parameters should be seen as aspirational, to be integrated as technology and resources allow, and harmonisation strategies are needed to standardise data collection and reporting across centres to ensure comparability.

For nAMD, comorbidities such as previous cataract surgery, previous vitrectomy, and fellow eye disease or status should also be collected as part of RWD standards (S19; 97%). In the case of DMO, additional parameters that should be collected are HbA1c measurements and current medications (S12; 97%), comorbidities such as hypertension, hypercholesterolemia, kidney disease, myocardial infarction, stroke, and peripheral vascular disease (S20; 97%), along with information about diabetes history, such as time since diagnosis, type of diabetes, mean HbA1c levels, trends in fasting glucose levels, and systemic therapies (e.g. insulin therapy and aGPL1) (S26; 96%). However, data collection of comorbidities and patient history requires access to EHRs, which can be complex and time-consuming. Therefore, collecting data regarding comorbidities and patient history should be encouraged if possible, but it may be considered more aspirational in time-constrained scenarios.

### Topic C: ideal clinical standards for RWD methodology

#### Recommendation 4

*All RWD studies should be transparent regarding the methodology and criteria used to define their study cohort to ensure the reproducibility, reliability, and credibility of the data*

Transparency regarding the methodology and criteria used to define study cohorts was considered by the authors to be the key takeaway from this topic (S28; 99%). Existing guidelines, such as STROBE [31] and RECORD [32], provide frameworks for transparent reporting of observational studies; however, to encourage transparency, all publications for RWD studies should include a flow chart to clarify how the core patient population was filtered and defined. Data and patient populations should also be reported in an unbiased way, clearly describing inclusion and exclusion criteria, reasons for attrition, and how any missing information was handled (S29 & 30; both 97%). This would help ensure that ophthalmic RWD is reproducible and reliable.

Moreover, there needs to be greater discussion around the differences between clinical trials and clinical practice. Many clinical trial protocols use treatment regimens, such as fixed monthly injections, that are not always feasible or reflective of routine clinical practice. As such, detailing real-world treatment patterns is essential. This should include recording the number of injections received versus the number of clinic visits, to assess whether patients are being treated according to “treat and extend” strategies. Capturing this information is essential, given that patients often receive fewer treatments in real-world settings compared to clinical trials[33]. Quantifying the discordance between intended and actual treatment can help identify and address these gaps in care, ultimately leading to improvements in treatment delivery and patient outcomes.

### Topic D: ideal patient-reported standards

#### Recommendation 5

*Patient-Reported Outcome Measures (PROMs) are critical to include in RWD research and should be collected at baseline, and then every 12 months to assess treatment impact and patient satisfaction*

#### Recommendation 6

*To ensure PROMs are regularly utilised, research needs to be undertaken to develop a rapid tool that can be easily used in clinical practice*

The consensus achieved for Topic D highlights the importance of collecting PROMs to facilitate more patient-centred care and enhance healthcare outcomes and experiences (S31–33; 95–96%). PROMs should be collected at baseline, and then every 12 months, to track changes over time. This would aid the identification of changes in a patient’s health status, highlight opportunities to improve eye care services, assess the effectiveness of interventions, and inform personalised treatment plans. Several validated PROMs measure the impact of visual impairment on quality of life; however, despite their recognised value, many are time-consuming to complete and analyse, posing challenges for clinicians and patients[34, 35]. The Visual Function Questionnaire (VFQ-3oo7) is a short, 7-item questionnaire, developed for use as a routine PROM. It has shown effectiveness in evaluating patients’ perceived vision-related health status, whilst minimising respondent burden, making it suitable for routine use [36]. Nevertheless, even with the availability of such tools, their integration into routine care remains limited [37]. Wider adoption may be achieved by integrating automated EHR prompts that remind clinicians when PROMs are due to be captured, or by asking patients to complete questionnaires digitally before appointments, with results synced to their records.

### Topic E: implementing & reporting consistent standards/frameworks

#### Recommendation 7

*More formal approaches need to be developed for educating, training, and supporting researchers, clinicians, and other stakeholders on the best practices for RWD collection*

#### Recommendation 8

*Continued evaluation and refinement of RWD data collection and methodology standards is needed to ensure best practice adapts to evolving healthcare landscapes and to support improved patient outcomes*

All statements in Topic E achieved high agreement, offering insight into how consistent standards and frameworks can be implemented for RWD collection and reporting. Providing education, training, and support to researchers, clinicians, and other stakeholders on best practices for RWD methodology (S34; 98%) was viewed by the SC as difficult, but highly important and achievable if dedicated forums and networks are established to facilitate this. Currently, processes for RWD training are often informal and ad hoc, relying on individual experience or local mentorship rather than standardised instruction. There is scope to develop formalised training programs, potentially through national and/or international professional bodies such as the Royal College of Ophthalmologists or the International Council of Ophthalmology. Structured education has been successfully implemented for RCTs, with dedicated courses covering the research process and design, data collection, analysis, and publication. Embedding RWD literacy within professional education systems through similar structured approaches may promote more consistent methodology, improve the quality of data generated, and provide more robust conclusions to inform clinical decision-making.

In addition, continued evaluation and refinement of RWD data collection and methodology standards are needed. S35, 36, and 38 achieved high levels of agreement (95–98%), which is reassuring considering that they were based on established standards and checklists from ICHOM and CONSORT. However, there is a gap between these established standards and their consistent implementation in practice. In this context, providing open access to the fully anonymised RWD dataset may promote transparent reporting, strengthen the validity and generalisability of results, and facilitate cross-study collaboration. However, open access must comply with local regulations, and controlled-access repositories may offer a pragmatic alternative where full public access is not feasible.

### Strengths and limitations

The strengths of this consensus come from the 244 responses that were received, with representation from experienced physicians across multiple geographic regions globally. The dissemination of the survey by The Ophthalmology Network ensured all respondents were appropriate for the study and experienced in research and in contributing to guideline development. A four-point Likert scale was chosen to elicit respondents’ opinions and avoid middle option bias. However, high agreement levels may suggest statements were constructed to achieve agreement (confirmation bias); but variations in the level of agreement with some statements by subgroup, e.g., years of experience and region, suggest genuine responses. There were limited responses from those located in Africa and Australia/Oceania, which hindered forming strong conclusions on the opinions of this demographic. This study also only included clinicians, which may limit the applicability of findings to other key stakeholder groups, such as payers and policymakers. Lastly, the use of the SC to disseminate the survey potentially limited the reach of the study to highly qualified participant groups from their networks, and in line with this, the high levels of agreement could indicate sample bias.

## Conclusion

This modified Delphi consensus highlights the unmet need for a standardised framework for ophthalmology RWD methodologies. The recommendations define a minimum dataset for baseline and follow-up assessments, promote single-point data entry systems to streamline collection, and emphasise routine PROM capture every 12 months to enhance consistency, outcome assessment, and patient-centred care. While these recommendations represent aspirational long-term goals, embedding RWD literacy within professional education systems represents a pragmatic initial step to facilitate wider adoption of standardised RWD collection practices. Overall, implementation of these recommendations has the potential to yield more meaningful and generalisable RWE to guide clinical decision-making and improve patient outcomes in ophthalmology.

## SUMMary

### What was known before

- Real-world data (RWD) is essential in ophthalmology to complement clinical trial evidence and guide routine practice.
- Available guidelines, such as those from ICHOM, define outcome measures that should be collected for macular degeneration and cataract surgery, but do not address the methodological inconsistencies that limit ophthalmology RWD studies.
- Lack of standardisation in data collection, reporting, and patient-reported outcomes reduces the reliability and comparability of RWD evidence.

### What this study adds

- This Delphi consensus achieved expert consensus on the need for a standardised framework for ophthalmology RWD methodologies.
- It defines a minimum dataset of clinical, treatment-related, and patient-reported measures to improve consistency and generalisability of RWD studies.
- It also provides recommendations on practical strategies, including single-point data entry systems, routine PROM collection, and formalised training, to support high-quality and reproducible RWD.

## Supplementary information

Supplementary Information

## Data Availability

All data relevant to the study are included in the article or uploaded as supplementary information on *Eye’s* website.
