# Supplementary material for: Defining a standards framework for ophthalmology real-world data methodologies through an expert-led Delphi consensus
Source: Eye (Lond). 2026 Apr 2;40(9):1342–50. doi: 10.1038/s41433-026-04424-1 (PMC13269761; doi:10.1038/s41433-026-04424-1)
Supplement: Supplementary file 1 — Supplementary Information [file 41433_2026_4424_MOESM1_ESM.docx]

**Supplementary information**

**Summary: Supplementary Figures 1–6:** Demographic profile of survey respondents, including role (S1), years of experience (S2), region (S3), institution type (S4), experience with real-world data (S5), and contribution to guideline development (S6).

**Figure S1.** Number of respondents per role.

**Figure S2.** Number of respondents by experience.

**Figure S3.** Number of respondents per region.

**Figure S4.** Number of respondents by type of institution worked in.

**Figure S5.** Number of respondents by experience of working with real-world data.

**Figure S6.** Number of respondents by contribution to guideline development.
